# Effect of Dietary Replacement of Fishmeal by Insect Meal on Growth Performance, Blood Profiles and Economics of Growing Pigs in Kenya

**DOI:** 10.3390/ani9100705

**Published:** 2019-09-20

**Authors:** Shaphan Y. Chia, Chrysantus M. Tanga, Isaac M. Osuga, Alphonce O. Alaru, David M. Mwangi, Macdonald Githinji, Sevgan Subramanian, Komi K. M. Fiaboe, Sunday Ekesi, Joop J. A. van Loon, Marcel Dicke

**Affiliations:** 1 Laboratory of Entomology, Wageningen University & Research, 6700AA Wageningen, The Netherlands; 2 International Centre of Insect Physiology and Ecology, Nairobi 00100, Kenya; 3 Department of Animal Sciences, Jomo Kenyatta University of Agriculture and Technology, Nairobi 00200, Kenya; 4 Non-Ruminant Research Institute (NRI), Kenya Agricultural and Livestock Research Organization (KALRO), Naivasha 20117, Kenya; 5 Non-Ruminant Research Institute (NRI). Kenya Agricultural and Livestock Research Organization (KALRO), Kakamega 50100, Kenya; 6 International Institute of Tropical Agriculture (IITA), BP 2008 (Messa), Nkolbisson, Yaoundé, Cameroon

**Keywords:** growing pigs, blood parameters, insect larval meal, alternative protein, animal feeds, cost benefit analysis, return on investment

## Abstract

**Simple Summary:**

Pig keeping is an important source of income but the high cost of fishmeal (FM), which is the main protein source in animal feeds, has hindered the sector from realizing its full potential. As an alternative, we investigated the suitability of a meal derived from black soldier fly larvae (BSFLM) as a protein source. Pigs were fed different diet types: Control (no BSFLM: 0% (T0)), 25% (T25), 50% (T50), 75% (T75) and 100% (T100) replacement of FM by BSFLM. Average daily feed intake, body weight gain and feed conversion ratio were not affected by the replacement of FM by BSFLM. Red or white blood cell parameters did not differ among diets, except for neutrophil counts, which were higher at T75 and T100 compared to T0. At T25, T75 and T100, pigs had lower platelet counts compared to pigs fed T0 and T50. Dietary BSFLM inclusion did not influence blood cholesterol levels. The cost–benefit ratio and return on investment were similar across diets. Our study shows that BSFLM is a suitable and cost-effective alternative to FM in pig feeds.

**Abstract:**

Pig production is one of the fastest growing livestock sectors. Development of this sector is hampered by rapidly increasing costs of fishmeal (FM), which is a common protein source in animal feeds. Here, we explored the potential of substituting FM with black soldier fly larval meal (BSFLM) on growth and blood parameters of pigs as well as economic aspects. At weaning, 40 hybrid pigs, i.e., crossbreeds of purebred Large White and Landrace were randomly assigned to five iso-nitrogenous and iso-energetic dietary treatments: Control (0% BSFLM and 100% FM (T0)), and FM replaced at 25% (T25), 50% (T50), 75% (T75) and 100% (T100) with BSFLM. Average daily feed intake (ADFI), average daily gain (ADG), body weight gain (BWG) and feed conversion ratio (FCR) were calculated for the whole trial. Hematological and serum biochemical parameters, the cost–benefit ratio (CBR) and return on investment (RoI) were evaluated. No significant effect of diet type was observed on feed intake and daily weight gain. Red or white blood cell indices did not differ among diets. Pigs fed T25, T75 and T100, had lower platelet counts compared to T0 and T50. Dietary inclusion of BSFLM did not affect blood total cholesterol, triglycerides, low-density lipoprotein and high-density lipoprotein. CBR and RoI were similar for the various diets. In conclusion, BSFLM is a suitable and cost-effective alternative to fishmeal in feed for growing pigs.

## 1. Introduction

Pig production is one of the fastest growing livestock sectors globally, with most of the growth occurring in the developing countries [1]. Pigs are of socio-economic value to smallholder farmers and provide a safety net in times of financial crisis [1]. A short breeding cycle, high fecundity, high feed conversion efficiency and increasing demand are major drivers of growth in this sector. In communities currently experiencing a shift from ruminant to non-ruminant livestock production, pig farming is becoming relevant [1,2]. However, expansion and profitability are constrained by increasing feed costs, especially the protein ingredients [1]. Feed costs represent 60%–70% of total costs in intensive pig production, which is especially due to costs of protein. In East Africa, major protein ingredients such as fishmeal (FM) and soybean meal are increasingly unavailable and expensive for smallholder farmers [3]. Consequently, farmers resort to alternative feed sources considered to be cheaper without knowledge of their influence on the physiological response and animal growth [2,4,5,6]. Considering the importance of pig production in livelihoods of smallholder farmers and the growing scarcity of protein ingredients as well as the environmental implications of producing these resources [7,8], dependence on FM and soybean meal is not sustainable [9].

Insects have high protein and fat content and have been considered promising high-quality feed components [10,11,12]. Insects could replace 25% to 100% of FM or soybean meal in feeds for livestock and aquaculture, depending on the insect, livestock and fish species [13]. The black soldier fly (BSF) *Hermetia illucens* L. (Diptera: Stratiomyidae) is distributed worldwide in the tropics and warm temperate regions [14]. BSF larvae feed on organic resources such as fruit remains, animal manure, vegetables and brewers’ spent grains [14,15,16,17,18,19,20,21,22] and convert these resources into high-quality insect protein and fat. In contrast to other dipteran species such as the house fly *Musca domestica*, BSF is not considered a pest and its larvae can reduce populations of harmful bacteria [23,24]. On a dry matter basis, BSF larvae contain 37% to 63% crude protein, 7% to 39% fat [11,25], 8% calcium, 1% to 2% phosphorus, 0.1% to 0.3% sodium and 0.4% to 1% magnesium [25,26,27,28]. BSF larval meal has been used as an ingredient in feed for fish [29,30,31,32,33,34,35,36], poultry [3,37,38] and pigs [39,40,41,42], with promising results. However, there is no exhaustive information on the influence of BSF larval meal (BSFLM) on performance and health response of growing pigs. The few studies on the inclusion of BSFLM in pig feeds have focused on piglets, replacing either FM or soybean meal with low (less than 10%) levels of BSFLM over short (10–40 days) periods of feeding [39,40,41,42]. The short experimental periods do not allow for a complete growth phase under the dietary supplementation and, therefore, do not reflect the common practice of pig feeding. In addition, higher levels of BSFLM inclusion in pig feed have not been assessed. In the present study, we subjected growing pigs to feeds with higher (25%–100%) dietary replacement levels of FM with BSFLM over an extended period (>60 days) of feeding, covering the complete pig grower phase and measured the effect of diet on performance and economics of pig feeding.

Hematological and biochemical parameters are affected by several factors, including diet [4]. Nutritional deficiencies and diseases influence clinical health status of animals [43]. In the case of nutritional deficiencies, blood profiling can provide an indication of the clinical health status as well as the extent to which dietary deficiencies impact physiological status of the animal, which allows farmers to adjust the diets of the animals to ensure that they receive adequate feed ingredients for optimal production [4,44,45]. BSF larvae represent a novel protein source in animal feed. Studies have reported unaffected growth performance, blood parameters, nutrient digestibility, gut morphology and histological features of piglets as well as gut antimicrobial potential of the inclusion of full-fat or partially defatted BSFLM in piglet feed at the rate of 4%–10% to replace soybean meal or FM [40,41,42], but there is little information on the effect of complete replacement of FM by full-fat BSFLM in pig feed. Utilization of insect meal as an animal feed ingredient is attracting interest from researchers because conventional feed ingredients are increasingly becoming unaffordable to resource-poor farmers due to rapidly rising costs. This requires us to search for alternative protein ingredients that can economically supplement conventional feed ingredients used in feed formulation without adverse effects on the health and performance of the animals. The inclusion of ingredients in feed formulation does not only aim at a balanced nutrient content for optimal growth performance, but also considers profitability of the production process [46]. Therefore, the present study aimed at evaluating the effect of substituting FM with BSFLM on (a) growth performance, (b) hematological and serum biochemical indices and (c) economic implications of BSFLM inclusion in growing pig diets.

## 2. Materials and Methods

The present study was conducted at the pig rearing facility of the Non-ruminant Research Institute (NRI) of the Kenya Agricultural and Livestock Research Organization (KALRO), Naivasha, Kenya. The general care and management of the animals followed accepted guidelines as described by the Federation of Animal Science Societies [47].

### 2.1. BSF Larval Meal (BSFLM) and Experimental Diets 

Diets were formulated to meet growing pig requirements [48]. Isonitrogenous and isoenergetic diets were prepared by replacing the FM content of a control diet (T0) at 25%, 50%, 75% and 100% (T25, T50, T75 and T100, respectively) with BSFLM, as experimental diets (Table 1). Maize meal, wheat pollard and rice polishing were included as energy sources. BSFLM and FM served as major protein sources. Vitamin and mineral premix, salt, limestone and bone meal served as vitamin and mineral sources (Table 1). FM, maize meal, rice polishing, salt, limestone, vitamin and mineral premix, lysine, methionine and bone meal were purchased from commercial animal feed retailers. BSF larvae were obtained from the International Centre of Insect Physiology and Ecology (*icipe*), reared following the BSF rearing procedure of the Animal Rearing and Containment Unit (ARCU), *icipe*. BSF larvae were reared on a mixture of brewers’ spent grains (BSGs) obtained from Kenya Breweries Limited (KBL). After harvest, larvae were sterilized by washing in warm water (84 °C) for 10 minutes and then oven-dried using a commercial stainless-steel fruit/vegetable/meat/fish drying machine model (CT-C-III Series hot air circulating drying oven, Henan Forchen Machinery Co., Ltd., Henan, China). The machine can dry 360 kg/batch of fresh insects for 2.5 h at 120 °C. Dried larvae were ground into larval meal using a hammer mill [39].

### 2.2. Proximate, Amino Acids and Mineral Composition of Experimental Diets

The dry matter content of formulated feed samples was gravimetrically determined after the loss of water. The samples were heated to 103 ± 2 °C for 3 h to constant weight. Ash content was determined by ignition of samples at 550 °C in a muffle furnace. Dried and ground samples were exposed to an electromagnetic scan in the absorbance mode using near infrared (NIR) spectroscopy (CROPNUTS, Nairobi, Kenya). The crude protein, fat, starch, oil, acid detergent fiber, neutral detergent fiber, sugar and digestibility (NCGD) values were determined following standard laboratory procedures and energy values were calculated [49,50].

Essential and non-essential amino acid contents of BSF larvae and experimental diets (Table 1) were analyzed by AMINOLab^®^ (Evonik Industries, Hanau, Germany) using an amino acid analyzer (Biochrom 30 plus, Biochrom Ltd. Cambridge, UK) [51,52,53,54,55]. Feed samples were homogenously ground with an Ultra Centrifugal Mill RETSCH-ZM 200 to pass through a 0.5 mm sieve. Finely ground samples were weighed using an analytical balance, display accuracy ±0.01 mg into 50 mL laboratory bottles, with thread of DURAN glass (Schott, Mainz, Germany), red polybutylene terephthalate (PBTP) caps with silicone/Teflon seal. Then, 25 mL of hydrochloric-phenol reagent was added to the sample in the bottles and the mixture was introduced into a thermostatically controlled heating oven (UT 6060 AR, Thermo Electron LED, Langenselbold, Germany) at 110 °C, with loose screw tops for one hour and then with tightened screw tops for 23 h to complete the sample hydrolysis. Methionine and cystine samples were prepared through performic acid oxidation procedures followed by the acid hydrolysis–sodium metabisulfite method [52,56]. The resulting hydrolysate solutions were then introduced into the amino acid analyzer and the sample aminograms were detected at 570 nm and 440 nm. Amino acid concentrations in the samples were determined in duplicate.

The mineral content of feed samples (Table 2) was analyzed by inductively coupled plasma-atomic emission spectrometry (ICP-OES; CROPNUTS, Nairobi, Kenya). Sample preparation involved microwave-assisted acid digestion. Aliquots of ground feed samples were transferred to a glass tube of the microwave system. Then, a mixture of nitric acid and hydrochloric acid was added to the sample and allowed to digest. The resulting solution was filtered into a volumetric flask and used for ICP-OES analysis to determine the following minerals: Boron, molybdenum, iron, copper, zinc, cobalt, manganese, sodium, sulphur, magnesium, potassium, phosphorus and calcium [57,58,59,60].

### 2.3. Experimental Animals and Housing

Before the commencement of the experiment, forty (20 male and 20 female) hybrid pig weaners, which consisted of a cross between purebred Large White and the Landrace with mean body weight of 18.25 ± 0.34 kg were sourced from Farmer’s Choice Limited, Nairobi, Kenya. The pigs were randomly assigned to five dietary treatments, each replicated four times per sex (four males and four females). Pigs were housed in concrete floor pens (3.65 m × 1.85 m) each containing two pigs (one male and one female). Each pen was provided with a one-sided self-feeder (1.80 m × 0.20 m × 0.18 m). Pigs were adapted to the pens for 14 days before the start of the experiment, during which they were fed a commercial starter feed. At the start of the experiment, each pen was labeled with a number and diet type while each animal was identified with a unique number by ear tattooing. A layer (~0.25 m thick) of dry wood shavings was carefully placed at one corner of the floor of each pen, which served as bedding for the pig and provided warmth. Pig pens were cleaned every day by scrubbing the floor using Teepee straight brooms (c27, Chandarana Foodplus, Nairobi, Kenya) and water. Each pen was provided with a nipple drinker fitted to the wall and the distance between the nipple and floor was adjusted as the pigs increased in height. Experimental animals were allowed ad libitum access to feed and water throughout the experiment.

### 2.4. Growth Performance 

Individual pig body weight was recorded on a weekly basis using a 150 kg × 500 g suspended weighing scale (Salter, model 235, Bilston, England), with the sides covered with a wire mesh to prevent uncontrolled exit of the animal. The entry gate of the weighing cage was opened sideward and the animal was led into a stable, non-moving floor after which the gate was closed. Once inside the cage, the animal was allowed to settle, and the weight value read on an analogue display screen above the weighing cage. After every reading, the pig was released from the weighing cage into its pen and the scale was moved to the next pen on two wheels fitted on the front end of the cage. On the day of weighing, pigs were only provided with feed after recording their body weight. The weekly body weights were used to calculate average daily weight gain (ADG). Feed offered to the pigs and unconsumed portions were weighed daily using a digital platform weighing scale (XK3190-A12, >300 kg, Gromy Scale Co., Ltd., Hangzhou, China) to calculate average daily feed intake (ADFI). The trial lasted nine weeks. Total body weight gain and feed consumed were used to calculate feed conversion ratio (FCR) for each dietary treatment.

### 2.5. Blood Characteristics

At the end of the growing pig phase, three randomly selected pigs from each dietary treatment were starved for 12 h, with access to drinking water only. After this period, two blood samples (5 mL each) were drawn from the peripheral ear vein using flashback blood collection needles and 9 mL vacutainer blood collection tubes (VP4082, Sunphoria Co., Ltd., Taipei, Taiwan). One of the samples was treated with the anticoagulant ethylene diamine tetra acetic acid (K2EDTA) and the other with serum clot activator. These samples were transported immediately to the laboratory for further analysis.

### 2.6. Hematological and Serum Lipid Parameters

A 5-part white blood cell (WBC) differential and complete blood cell count was performed using the automated IDEXX ProCyte DxTM Hematology analyzer (IDEXX, Westbrook, ME, USA) by laser flow cytometry, optical fluorescence and laminar flow impedance. Each ethylene diamine tetra acetic acid (EDTA)-anticoagulated whole blood sample was mixed thoroughly for seven minutes on a sample rocker. Once the processing was over, the sample details were entered and the type of analysis to be carried out was indicated. Thereafter, the blood samples were loaded into the analyzer and automatically run to generate the following parameters: Red blood cells (RBC), hemoglobin concentration (Hb), hematocrit (Hct), mean corpuscular volume (MCV), mean corpuscular hemoglobin (MCH), mean corpuscular hemoglobin concentration (MCHC), red cell distribution width (RDW), platelet count, total white blood cell count (WBC), neutrophil percentage, lymphocyte percentage, monocyte percentage, eosinophil percentage and basophil percentage. The WBC differential counts were qualitatively verified through Romanowsky-stained thin blood smear examination using a light microscope at the oil immersion objective (100×). Clotted blood samples were centrifuged at 4000 revolutions per minute (rpm) for 10 minutes. The serum lipoprotein (HDL), triglyceride and low-density lipoprotein (LDL) levels in the samples were measured on an automatic Cobas Integra 400 plus Chemistry Analyzer (Roche, Rotkreuz, Switzerland) using enzymatic colorimetry.

### 2.7. Economic Analysis

Two key parameters, the cost–benefit analysis (CBA) and return on investment (RoI) [3] were used to evaluate the economic implication of replacing FM in pig diets with BSFLM. The cost–benefit ratio (CBR), as an indicator in CBA, was used to summarize the economic value of replacing FM with BSFLM in pig diets. Here, it was assumed that all other costs of production were constant for all dietary treatments, except the cost of the feed, which was considered in the CBR and RoI calculations. Feed costs were calculated from the ingredient prices based on quantities of each item incorporated in the dietary treatments. The total revenue from the pigs was estimated by considering 3.0 US $/kg of pig’s live body weight, assumed to represent all the benefits that would be received from the production. The ratio between the production revenue and the production cost represents the CBR. A CBR value greater than one suggests that the benefits of the production exceeded the production costs and vice versa. RoI is a measure of gain/loss generated from an investment relative to the money invested. The higher the RoI value the better the returns of the project under consideration [3,61].

### 2.8. Statistical Analysis

General linear modeling was used to assess the effect of diet on growth performance, blood parameters and economic parameters of pigs fed BSFLM-based diets and a control diet over a nine-week period. Collinearity of variables was checked to obtain independent covariates. The model for each analysis included all independent variables, which were removed one by one until the Akaike information criterion (AIC) was at a minimal level. For growth performance, diet, sex and their interaction effect were included for the analysis of ADG, body weight gain (BWG) and final body weight (FBW). For weekly body weight (BW), diet, sex, time (week) and their interaction effects were included in the model. Two pigs (female and male) per replicate were provided with feed in the same trough. Hence, ADFI analysis by sex was not possible. Three randomly selected pigs per dietary treatment were used for the blood parameter assessment, with diet as the explanatory variable. Similarly, the economic analysis was based on ADFI, hence diet was included as the explanatory variable in the model. Mean effects were considered statistically significant at *p* < 0.05, with a least significant difference test (LSD) as the post-hoc test. All statistical analyses of the data were implemented using R software (version 3.5.1).

### 2.9. Ethical Approval

Ethical approval for the study was provided by the Institutional Animal Care and Use Committee (IACUC) of Kenya Agricultural and Livestock Research Organization (KALRO)—Veterinary Science Research Institute (VSRI); approval Code No.: KALRO-VSRI/IACUC019/30082019.

## 3. Results

### 3.1. Growth Performance and Feed Conversion

Pigs readily accepted experimental diets and no mortality was recorded. Neither diet nor sex affected initial weight, final body weight (FBW) or average daily weight gain (ADG); the interaction between diet and sex was also not significant (Table 3).

**Figure 1:**
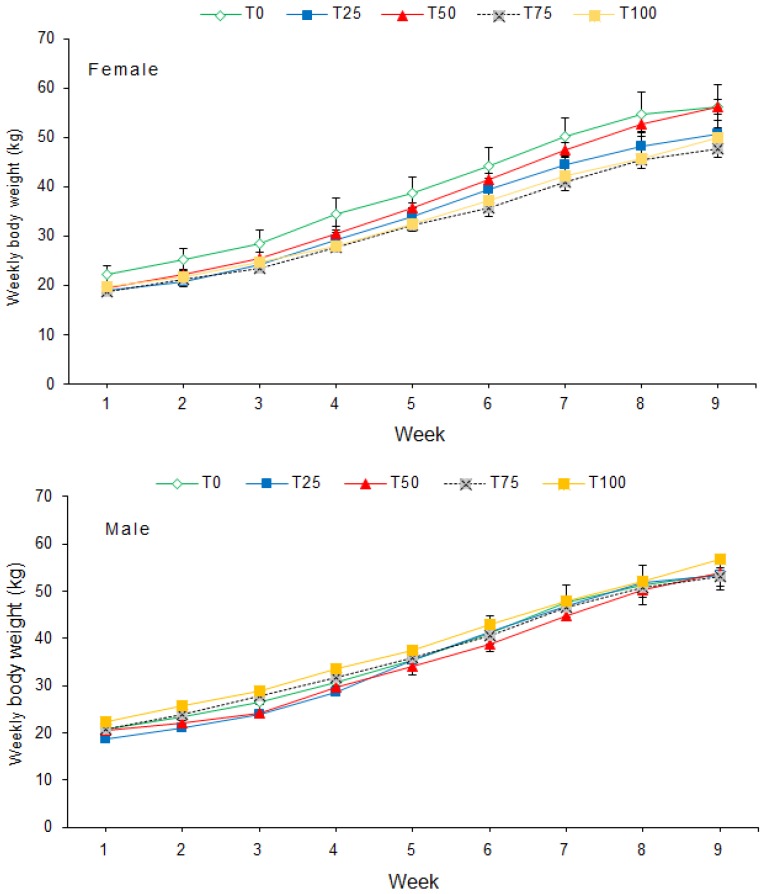
Mean (±SE) weekly body weight of pigs fed BSFLM-based diets and a control diet. BSFLM = black soldier fly larval meal. T0 = 0% (control), T25 = 25%, T50 = 50%, T75 = 75% and T100 = 100% levels of replacement of fishmeal with BSFLM. For each diet, four males and four females were investigated.

Weekly body weight (BW) differed significantly among diets (*p* < 0.001) and sexes (*p* = 0.005). BW increased significantly (*p* < 0.001) from week 1 to week 9 for male and female pigs (Figure 1). The interaction between diet and sex on BW was significant (*p* < 0.001). There was no significant interaction between diet and week (*p* = 0.110) or between sex and week on BW (*p* = 0.388). Furthermore, the interaction between diet, sex and week on BW was also not significant (*p* = 0.345). 

**Figure 2:**
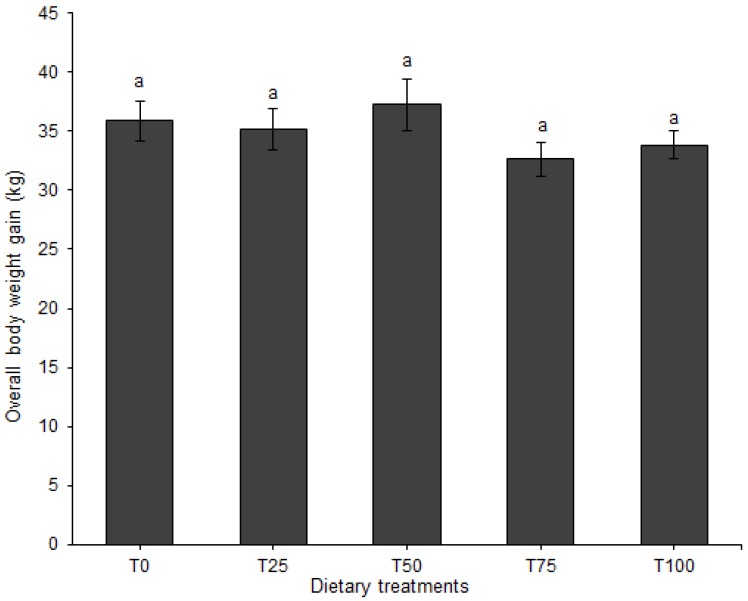
Body weight gain (mean ± SE) of pigs fed BSFLM-based diets and a control diet for the whole trial. Bars followed by the same letter are not significantly different: *p* < 0.05, general linear model (GLM, least significant difference test (LSD)). BSFLM = black soldier fly larval meal. T0 = 0% (control), T25 = 25%, T50 = 50%, T75 = 75% and T100 = 100% levels of replacement of fishmeal with BSFLM. For each diet, four males and four females were investigated. Data for female and male pigs pooled together because there is no significant effect of sex. *n* = 8 per bar.

Body weight gain for the entire experimental period (BWG) was neither affected by diet (*p* = 0.351; Figure 2) nor by sex (*p* = 0.486) and neither was the interaction between diet and sex (*p* = 0.340).

**Figure 3:**
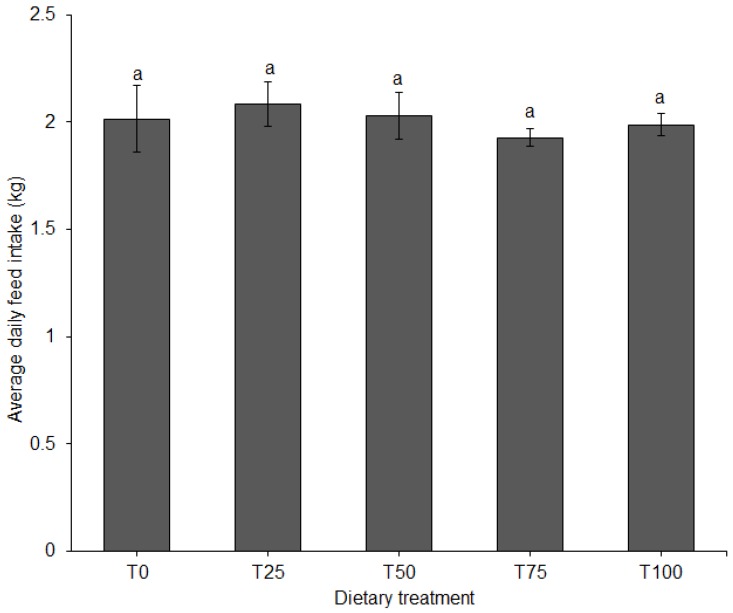
Average daily feed intake (±SE) in pigs fed BSFLM-based diets and a control diet. Bars with the same letters are not significantly different: *p* < 0.05, general linear model (GLM), BSFLM = black soldier fly larval meal. T0 = 0% (control), T25 = 25%, T50 = 50%, T75 = 75% and T100 = 100% levels of replacement of fishmeal with BSFLM. *n* = 8 per bar.

**Figure 4:**
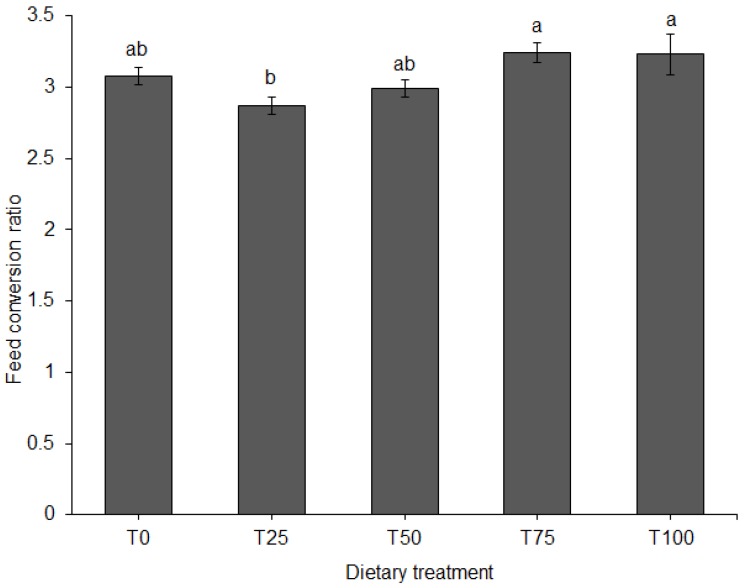
Feed conversion ratio (mean ± SE) for pigs fed BSFLM-based diets and a control diet. Bars with different letters are significantly different: *p* < 0.05; general linear model (GLM), LSD. BSFLM = black soldier fly larval meal. T0 = 0% (control), T25 = 25%, T50 = 50%, T75 = 75% and T100 = 100% levels of replacement of fishmeal with BSFLM. *n* = 8 per bar.

ADFI did not differ among diets (Figure 3). FCR differed significantly among diets (*p* = 0.011). When fed T75 or T100, FCR of the pigs was significantly higher compared to T25 (Figure 4).

### 3.2. Hematological and Serum Lipid Parameters 

Red blood cell indices did not differ among dietary treatments (Table 4). Hb at T25-T100 was within the normal range, whereas at T0, Hb was slightly below the normal range. MCHC values at T25–T100 were below the normal range compared to T0. Platelet count differed significantly among diets (Table 4). At T0, platelet count was and within the normal range compared to treatments T25, T75 and T100 (Table 4).

WBC count did not differ among pigs fed with different diets (*p* = 0.463). At T0 and T50, WBC counts were within the normal range, whereas the WBC counts of pigs from the other dietary groups were slightly above the normal range (Table 5). Diet significantly affected neutrophil counts (Table 5). At T75 and T100, neutrophil counts were significantly higher and within the normal range compared to T0 and T25 (Table 5). Lymphocyte counts did not differ among diets (Table 5). At T100, lymphocyte count was within the normal range, whereas at T0–T75 the values were above the normal range. Monocyte, eosinophil and basophil counts did not differ among diets and were all within the normal range (Table 5). Serum lipid parameters did not differ among diets. All serum lipid parameters investigated were within the normal range (Table 5).

### 3.3. Economic Analyses of the Inclusion of Black Soldier Fly Larva Meal in Pig Diets

Replacing fishmeal by BSFLM in pig diet did not affect the profit accrued from the sale of pigs (Table 6). The cost–benefit ratio and return on investment did not differ among diets (Table 6). 

## 4. Discussion

In Africa, to the best of our knowledge, the current study is the first to report the positive impact of non-defatted BSF larvae as a protein-rich ingredient in pig feeds. Protein is an important component of animal feeds required for growth and development. The source of protein is crucial because it affects availability and utilization of the essential amino acids [62]. The crude protein (CP) content of BSF larvae largely depends on the substrate used to rear the larvae and varies from 39% to 44% [63], which is comparable or superior to that of the commonly used soybean and FM [3]. BSFLM is a suitable ingredient in pig feed [13] and feeds containing BSFLM are as palatable as those containing soybean meal [39]. Our results agree with earlier studies in which partially defatted or full-fat BSF inclusion levels of only 4%–10% in partial replacement of either FM or soybean meal did not result in a significant difference in growth performance of the piglets [40,41,42], when compared to the conventional FM /soybean diets. The present study shows that much higher replacement levels, up to 100% were acceptable and well tolerated by the pigs. No significant decline in growth parameters or mortality was recorded. Acceptability and suitability of BSFLM has also been successfully recorded for fish and chicken [33,64]. Thus, our study shows that BSFLM can successfully replace FM as a sustainable protein-rich ingredient in growing pig diet as reflected in the growth performance and feed conversion rate, which were similar for all the treatment and control diet groups of pigs. Rejection of feed due to texture, palatability or inclusion of BSFLM was not observed, which is in line with the observations by Ramos-Elorduy et al. [65] for broiler chickens.

In animal production, ADG is a critical index of growth performance [66]. We did not observe a significant effect of dietary treatment on ADG of the pigs, which is a clear indication of adequate nutrient supply by the different formulated diets. Tolerance of insect-based protein-rich diets for pigs has been documented in other studies [67,68,69,70,71]. In India, silkworm meal was used to completely replace FM in the diet of growing and finishing pigs without altering carcass and meat quality and blood parameters [67,68,69]. Similarly, in Nigeria, feeding early weaned pigs with a 3:1 mixture of dried rumen content and maggot meal in the diet replacing 10% wheat offal did not have adverse effects on performance [72]. The inclusion of 10% and 15% of defatted BSFLM in the diet of growing quails (from 10 to 28 d of age) led to comparable production performances and carcass traits with those of quails fed conventional soybean meal and oil-based diets [73]. The suitability of BSFLM for the growing pigs in our experiments can be attributed to the high level of digestibility, which is consistent with other studies on pigs fed with BSF larvae [39], and broilers fed with housefly pupal meal [74].

In contrast to the current study, Newton et al. [70] reported that a complete replacement of dried plasma with BSF pre-pupal meal in the diet of early weaned pigs reduced performance of the pigs by 3%–13%. Poor performance of weaner pigs fed on pre-pupal meal might be attributed to the higher chitin levels in the pre-pupae than in larvae as used in our study, which has been reported to contribute to decreased digestibility resulting in reduced nutrient utilization and growth performance in animals when higher substitution rates are used [70,75]. Low digestibility of BSF pre-pupal protein in animal feeds is also supported by Bosch et al. [11], who attributed this to higher cuticular protein-sclerotization in the pupae. The similarity in feed intake and average daily weight gain recorded for all the treatment groups in the current study can be attributed to utilization of the 5^th^ instar larval meal instead of the pre-pupal meal, which contributes sufficient nutrients in the diets with high level of digestibility. BSFLM has also been shown to be of good nutrient composition for reptiles [76].

In the present study, the values of the hematological parameters RBC, Hb, Hct, MCH, MCV, MCHC and RDW for pigs fed BSFLM were not significantly influenced by the replacement levels. The values for RBC, Hb, Hct, MCH and RDW fell within the physiological range for pigs, which is a clear indication of a good health status of the animals, implying that the quality of the test diets was adequate to maintain good health of the pigs. Dietary replacement of FM with BSFLM at the rates of 25%, 50%, 75% and 100% in pig diet improved RBC, Hb, Hct and RDW, which had higher values compared to the control FM diet group of pigs. The RBC counts and Hb concentration in blood increased to a level of 37% and 35.3%, respectively, at a FM replacement level of 50% with BSFLM. These results are consistent with the reports by Marono et al. [77] and Loponte et al. [78], who reported that dietary BSFLM inclusion positively affected the blood profile of laying hens and Barbary partridges, in terms of higher globulin levels. Our results may be attributed to high digestibility of insect-based protein and high levels of minerals such as iron, which is required for the formation of haemoglobin in the pigs. The higher the haemoglobin concentration, the better the oxygen circulation in the body, hence, better performance of the animal [79]. 

The results of the present study show that the composition of the various treatments significantly affected platelet count in pigs. The replacement of FM with BSFLM at rates of 25%, 75% and 100% in the feed was associated with significantly lower blood platelet counts out of the normal range than observed with 50% replacement of FM with BSFLM or with the control diet without BSFLM. This implies that diet composition in the present study significantly influenced the developing hematological system with some unknown factors suppressing the normal developmental increase in platelet counts in growing pigs. Low platelet concentration implies that blood clotting might be impaired, resulting in blood loss in case of injury [4]. According to Martin et al. [80], bleeding time largely depends on both platelet counts and mean platelet volume.

The largely similar WBC count obtained in this study implies that the ability of the pigs to respond to and eliminate infection was not compromised with the inclusion of BSFLM to replace FM in the diets. The normal monocyte levels may indicate that the pigs did not react to any infections during the experimental period. Furthermore, the similarity in basophil levels indicates that the pigs showed no hypersensitivity reaction to the inclusion of BSFLM in diets while the normal levels of eosinophils might indicate that the pigs did not suffer from parasitic infections during the experimental period [81,82]. However, higher (75% and 100%) levels of replacement of FM with BSFLM significantly improved the neutrophil count to the normal physiological range compared to the control FM diet. Neutrophils play an important role in immune responses, especially in wound healing through microbial sterilization and macrophage attraction [83]. BSF larval fat contains medium-chain saturated fatty acids with antimicrobial properties [41,84]. For instance, lauric acid has been identified as the most predominant medium chain saturated fatty acid found in BSF larvae. It has been shown that inclusion of coconut oil, which contains medium-chain saturated fatty acids in rabbit feeds significantly increases leucocytes and neutrophil counts [85]. The increase in neutrophil count in the present study could be an indication of the antimicrobial response in pigs fed high BSFLM-based diets. According to Ahlante et al. [85], increased mobilization of leucocytes and neutrophils in animals fed with coconut oil-based feed resulted from the stimulation of the pluripotent haemopoietic stem cells from which leucocytes are produced in the presence of a growth inducer and differentiation inducer, which are proteins. Neutropenia is a consequence of reduced neutrophil and leucocyte levels, which leads to reduced body immunity. Therefore, the inclusion of BSFLM in pig feed is highly recommended due to the valuable nutrients available to the growing pigs. The lymphocyte counts of pigs fed 100% FM diet, 25%, 50% and 75% BSFLM diets were higher and out of the normal range. This implies that these diets might have stimulated both cellular and humoral immune response systems of the pigs to protect against intracellular and extracellular pathogens such as *Mycobacterium*, *Listeria*, *Brucella*, *Pasteurella* or *Salmonella*, viruses and fungi [86].

The replacement of FM by BSFLM up to 100% did not affect the serum biochemical indices that are indicators of pig health. It is worth noting that although the use of hematological and biochemical indices have been considered as a fast means of assessing nutritional and health status of farmed animals, this has rarely been used in pig veterinary practices [42]. The observation that replacing FM by BSFLM does not affect serum biochemical indices in pigs supports the conclusion that BSFLM could form the basis of a valuable component in grower pig diets.

The cost–benefit analysis, which assesses whether an investment is sound and if—and by how much—profits outweigh costs, allows for comparing costs and benefits of alternative investments [87] as in the present study. The similarity in results obtained for the ‘control’ (100% FM diet) and BSFLM-based diets indicate that BSFLM is not only a valuable component of pig feed from a performance perspective but also from an economic perspective. This supports the need to further investigate the economic prospects of using BSFLM in large-scale pig feed formulation and feeding programs. A key advantage of insects as a feed ingredient, especially the BSF larvae over other conventional protein sources is their ability to convert waste into high-value biomass and closing nutrient cycles as they reduce pollution and costs of managing organic waste [88].

## 5. Conclusions

Dietary replacement of FM up to 100% with full-fat BSFLM did not adversely affect growth, blood characteristics or economic parameters. Although some changes in blood cell counts were observed, values were largely similar among diets. Pigs did not show visual signs of illness or abnormal behavior. Moreover, serum biochemical parameters were all within normal range for pigs. The present study indicates that a complete replacement of FM with full-fat BSFLM as an ingredient in growing pig feed is feasible, with reduced predisposition to heart diseases associated with high total cholesterol and LDL. Cost–benefit analysis results of the present study indicate that the inclusion of BSFLM in pig feed is a worthwhile investment for pig farmers. Finally, there is little evidence to suggest that adverse health effects should be expected in pigs following BSFLM consumption. Further studies are required to assess the effect of feeding BSFLM to pigs on meat sensory attributes and consumer perceptions.

## Figures and Tables

**Table 1:** Ingredients, composition of black soldier fly larval meal and experimental diets.

Parameter	BSFLM	Dietary Treatment				
		T0	T25	T50	T75	T100
Ingredient (kg)						
Maize meal	-	12.0	14.0	14.0	14.0	15.0
Wheat pollard	-	52.0	35.0	33.0	35.2	34.2
Rice polishing	-	22.0	30.5	32.0	29.8	28.3
Fishmeal	-	10.0	7.5	5.0	2.5	-
BSFLM	-	-	9.0	12	14.5	18.5
Lysine	-	1.0	1.0	1.0	1.0	1.0
Methionine	-	1.0	1.0	1.0	1.0	1.0
Salt	-	0.2	0.2	0.2	0.2	0.2
Bone meal	-	0.8	0.8	0.8	0.8	0.8
Limestone	-	0.8	0.8	0.8	0.8	0.8
Vitamin and mineral premix ^a^	-	0.2	0.2	0.2	0.2	0.2
Dry matter (DM; %)	94.9	92.6	94.0	92.4	93.2	94.0
Crude protein (% DM)	46.6	15.4	15.3	15.0	15.7	14.8
Essential amino acids (% DM)						
Lysine	2.9	1.0	0.8	0.9	1.0	0.9
Methionine	0.8	0.5	0.4	0.5	0.5	0.5
Methionine + Cystine	1.1	0.7	0.6	0.8	0.7	0.7
Isoleucine	1.8	0.5	0.5	0.5	0.5	0.5
Leucine	2.9	1.0	1.1	0.9	1.0	0.9
Threonine	1.7	0.5	0.5	0.5	0.5	0.5
Phenylalanine	2.2	0.6	0.6	0.5	0.6	0.6
Valine	2.6	0.7	0.7	0.7	0.7	0.7
Arginine	2.2	0.9	0.9	0.8	0.8	0.8
Histidine	1.3	0.3	0.4	0.3	0.3	0.3
Nonessential amino acids (% DM)						
Alanine	3.0	0.8	0.9	0.8	0.9	0.8
Aspartic acid	3.9	1.1	1.2	1.1	1.1	1.0
Cystine	0.4	0.3	0.3	0.2	0.2	0.2
Glutamic acid	4.9	2.3	2.3	2.0	2.1	2.0
Glycine	2.5	0.8	0.8	0.7	0.8	0.7
Proline	2.4	0.8	0.9	0.8	0.8	0.8
Serine	1.8	0.6	0.6	0.6	0.6	0.6

BSFLM = black soldier fly larval meal. T0 = 0% (control), T25 = 25%, T50 = 50%, T75 = 75% and T100 = 100% levels of replacement of fishmeal with BSFLM. DM = dry matter. Amino acid values are means of duplicate analyses. ^a^ Premix provided per kg diet: Vitamin A 6,000,000 IU; Vitamin D_3_ 1,000,000 IU; Vitamin E 5000 IU; Vitamin K_3_—KASTAB 3000 mg; Vitamin B_2_ —riboflavin 4500 mg; Vitamin B_3_—nicotinic acid 22,000 mg; Vitamin B_5_—pantothenic acid 16,000 mg; Vitamin B_6_ —pyridoxine 2250 mg; Vitamin B_9_—folic acid 350 mg; Vitamin H—biotin 50 mg, Vitamin B_12_—cobalamin 22 mg; choline chloride 150,000 mg; antioxidant 125,000 mg; iron (Fe) 40,000 mg, manganese (Mn) 40,000 mg; zinc (Zn) 100,000 mg, copper (Cu) 25,000 mg; iodine (I) 1000 mg, cobalt (Co) 250 mg, selenium (Se) 100 mg.

**Table 2:** Mineral and proximate composition of experimental diets.

Parameter	Dietary Treatment				
	T0	T25	T50	T75	T100
Boron (ppm)	4.2	2.8	2.3	2.7	3.1
Molybdenum (ppm)	1.3	1.1	0.5	1.0	1.2
Iron (ppm)	750.5	731.0	632.6	518.4	529.2
Copper (ppm)	22.6	501.1	80.5	16.7	22.8
Zinc (ppm)	121.4	110.6	114.1	102.3	112.6
Cobalt (ppm)	3.4	0.5	0.4	0.5	0.4
Manganese (ppm)	295.4	257.0	264.1	243.0	222.9
Sodium (ppm)	2439.8	1648.6	1097.8	1138.8	998.9
Sulphur (%)	0.4	0.6	0.3	0.3	0.3
Magnesium (%)	0.8	0.7	0.7	0.6	0.6
Potassium (%)	0.9	0.9	0.9	0.9	0.9
Phosphorus (%)	0.9	0.7	0.8	0.8	0.7
Calcium (%)	2.5	2.8	2.1	1.7	1.8
Sugar (%)	4.5	5.1	6.2	6.5	8.7
Starch (%)	29.2	25.3	27.6	20.7	26.9
Ash (%)	10.5	10.6	10.1	10.1	8.9
Acid detergent fiber (%)	15.3	19.0	17.0	20.7	15.4
Neutral detergent fiber (%)	37.7	42.8	41.6	47.0	39.7
Digestibility (NCGD) (%)	79.3	73.5	75.0	70.0	76.9
Oil (%)	5.0	8.1	8.9	9.8	12.2
Net energy (MJ/kg)	9.5	9.8	10.5	9.9	12.0

TT0 = 0% (control), T25 = 25%, T50 = 50%, T75 = 75% and T100 = 100% levels of replacement of fishmeal with BSFLM. NCGD = neutral cellulase gammanase digestibility, ppm = parts per million.

**Table 3:** Effects of dietary BSFLM inclusion on the growth performance of growing pigs.

Parameter	Sex	Dietary Treatment					*p* Value (GLM)		
		T0	T25	T50	T75	T100	Diet	Sex	Diet × Sex
Initial weight, kg	F	19.5 ± 1.73	16.8 ± 0.63	17.1 ± 0.66	17.0 ± 0.87	18.5 ± 0.71			
	M	18.8 ± 1.56	17.1 ± 0.85	18.5 ± 0.89	18.5 ± 0.79	20.8 ± 0.83			
	Overall	19.1 ± 1.09	17.0 ± 0.49	17.8 ± 0.57	17.8 ± 0.61	19.6 ± 0.66	0.080	0.152	0.628
Final weight, kg	F	56.3 ± 4.3	50.8 ± 2.65	56.3 ± 1.45	47.8 ± 1.76	50.0 ± 1.43			
	M	53.8 ± 3.56	53.5 ± 3.68	53.9 ± 4.90	53.0 ± 1.97	56.9 ± 0.99			
	Overall	55.0 ± 2.63	52.1 ± 2.16	55.1 ± 2.41	50.4 ± 1.58	53.4 ± 1.53	0.474	0.295	0.395
Average daily gain, kg	F	0.61 ± 0.04	0.56 ± 0.04	0.62 ± 0.04	0.50 ± 0.03	0.54 ± 0.03			
	M	0.59 ± 0.04	0.57 ± 0.04	0.62 ± 0.04	0.57 ± 0.04	0.59 ± 0.03			
	Overall	0.60 ± 0.03	0.57 ± 0.02	0.62 ± 0.02	0.53 ± 0.02	0.57 ± 0.02	0.126	0.408	0.641

BSFLM = black soldier fly larval meal. General linear model (GLM) *p* < 0.05. For each diet, *n* = 4; F = female; M = male. Overall = data for female and male pigs pooled together. T0 = 0% (control), T25 = 25%, T50 = 50%, T75 = 75% and T100 = 100% levels of replacement of fishmeal with BSFLM.

**Table 4:** Effects of dietary BSFLM inclusion on red blood cell indices and platelet count of growing pigs.

Parameter	Dietary Treatment					*p* Value (GLM)	Normal Range
	T0	T25	T50	T75	T100		
RBC (×10^12^ /L)	5.4 ± 2.3	7.0 ± 0.2	7.4 ± 0.1	6.8 ± 0.6	6.9 ± 0.1	0.725	5.0–8.00
Hb (g/dL)	10.2 ± 4.3	13.3 ± 0.2	13.8 ± 0.4	12.8 ± 1.0	13.5 ± 0.3	0.719	10.7–16.7
Hct (%)	35.5 ± 15	48.0 ± 1.0	47.6 ± 1.7	45.2 ± 3.2	47.0 ± 2.1	0.704	32.0–50.0
MCV (fl)	64.4 ± 1.7	69.1 ± 3.0	64.4 ± 3.2	66.5 ± 1.8	68.1 ± 2.4	0.593	50.0–68.0
MCH (pg)	19.3 ± 0.5	19.1 ± 0.8	18.6 ± 0.8	18.8 ± 0.4	19.6 ± 0.3	0.803	17.0–21.0
MCHC (g/dL)	30.0 ± 1.5	27.8 ± 0.1	28.9 ± 0.2	28.3 ± 0.8	28.8 ± 0.7	0.449	30.0–34.0
RDW (%)	20.5 ± 1.7	21.8 ± 0.2	22.0 ± 0.5	21.6 ± 1.0	22.2 ± 0.4	0.706	15.0–27.0
Platelet (K/uL)	382 ± 7.0 ^1 a^	209 ± 49 ^c^	328 ± 33 ^ab^	229 ± 27 ^bc^	251 ± 28 ^bc^	0.042	300–700

BSFLM = black soldier fly larval meal. RBC = red blood cell, Hb = hemoglobin, Hct = hematocrit, MCV = mean corpuscular volume, MCH = mean corpuscular hemoglobin, MCHC = mean corpuscular hemoglobin concentration, RDW = red cell distribution width. ^1,a,b,c^ *n* = 2. Means (± SE) within a row followed by different letters are significantly different: *p* < 0.05, general linear model (GLM), LSD. T0 = 0% (control), T25 = 25%, T50 = 50%, T75 = 75% and T100 = 100% levels of replacement of fishmeal with BSFLM. For each diet, three pigs were investigated.

**Table 5:** Effects of dietary BSFLM inclusion on white blood cell and serum biochemical indices of growing pigs.

Parameter	Dietary Treatment					*p* Value (GLM)	Normal Range
	T0	T25	T50	T75	T100		
WBC (k/L)	17.3 ± 1.6	24.5 ± 2.7	20.6 ± 2.5	24.0 ± 3.6	24.8 ± 5.0	0.463	11.0–22.0
Differential count (%)							
Neutrophils	24.2 ± 1.0 ^c^	24.7 ± 0.4 ^c^	27.1 ± 1.1 ^bc^	29.9 ± 2.9 ^ab^	31.8 ± 0.4 ^a^	0.019	28.0–51.0
Lymphocytes	69.4 ± 1.4	66.8 ± 0.8	64.8 ± 1.2	62.4 ± 4.0	60.9 ± 1.2	0.092	39.0–62.0
Monocytes	3.5 ± 0.7	5.6 ± 0.5	4.8 ± 0.9	4.1 ± 0.8	4.3 ± 1.2	0.496	2.00–10.0
Eosinophils	2.8 ± 0.5	3.0 ± 0.1	3.3 ± 0.3	3.5 ± 0.3	2.9 ± 0.5	0.684	0.50–11.0
Basophils	0.13 ± 0.03	0.08 ± 0.01	0.07 ± 0.03	0.15 ± 0.03	0.10 ± 0.0	0.585	0.00–2.00
Blood serum indices (mmol/L)							
Total Chol	1.90 ± 0.16	2.11 ± 0.13	2.11 ± 0.20	2.50 ± 0.12	2.19 ± 0.12	0.185	1.68–5.81
Total Trig	0.69 ± 0.08	1.03 ± 0.23	0.99 ± 0.17	0.89 ± 0.03	0.89 ± 0.07	0.479	0.11–1.13
LDL	0.67 ± 0.11	0.88 ± 0.08	0.75 ± 0.12	0.71 ± 0.11	0.65 ± 0.04	0.50	<3.00
HDL	1.23 ± 0.09	1.23 ± 0.07	1.37 ± 0.09	1.77 ± 0.23	1.53 ± 0.09	0.066	>1.00

BSFLM = black soldier fly larval meal. WBC = white blood cell. Means within a row followed by different letters are significantly different: *p* < 0.05, general linear model (GLM), LSD. BSFLM = Black soldier fly larval meal. Chol = cholesterol, Trig = triglycerides, LDL = low density lipoproteins, HDL= high density lipoproteins. T0 = 0% (control), T25 = 25%, T50 = 50%, T75 = 75% and T100 = 100% levels of replacement of fishmeal with BSFLM. For each diet, three pigs were investigated.

**Table 6:** Economic analyses of replacing fishmeal by BSFLM in growing pig diets.

Parameter	Dietary Treatment					*p* Value (GLM)
	T0	T25	T50	T75	T100	
Cost feed (US $/kg)	0.5	0.52	0.52	0.51	0.51	-
Cost of protein ingredient in feed (%)	0.24	0.31	0.31	0.3	0.3	-
Total feed consumed (kg/pig)	126.6	128.5	127.9	121.6	126	-
Cost of feed consumed per pig (US$), C_p_	62.89	67.29	66.14	61.97	64.43	-
Final body weight of pig (kg)	55.0 ± 2.63	52.1 ± 2.16	55.1 ± 2.41	50.4 ± 1.58	53.4 ± 1.53	0.474
Amount at final weight (US$/live weight), S_p_	165.0 ± 7.89	156.4 ± 6.49	165.2 ± 7.22	151.7 ± 4.42	160.1 ± 4.58	0.477
Profit, P_r_	102.1 ± 7.89	89.1 ± 6.49	99.0 ± 7.22	89.7 ± 4.42	95.9 ± 4.58	0.499
Cost–benefit ratio, CBR	2.6 ± 0.13	2.3 ± 0.10	2.5 ± 0.11	2.5 ± 0.07	2.5 ± 0.07	0.318
Return on investment, RoI	162.4 ± 13	132.4 ± 9.64	149.7 ± 11	144.8 ± 7.13	148.8 ± 7.11	0.318

BSFLM = black soldier fly larval meal. (-) values were not calculated. Cost (US $/kg) of protein ingredients used in the dietary treatments: Fishmeal (*Rastrineobola argentae*) = 1.20; BSFLM = 0.85. Live weight of pig = 3.00 US$/kg; P_r_ = S_p_−C_p_; CBR = S_p_/C_p_; RoI = P_r_/C_p_ × 100. Final body weight, S_p,_ P_r,_ CBR and RoI are expressed as mean ± standard error of the mean. General linear model (GLM), *p* < 0.05. T0 = 0% (control), T25 = 25%, T50 = 50%, T75 = 75% and T100 = 100% levels of replacement of fishmeal with BSFLM. For each diet, eight pigs were investigated. C_p_ = Cost price of feed consumed per pig, S_p_ = Selling price per pig (amount at final weight), P_r_ = Profit, CP = Crude protein.
